# ARFGEF2-Related Periventricular Nodular Heterotopia: A Case Report and Literature Review

**DOI:** 10.3390/neurosci7030063

**Published:** 2026-05-21

**Authors:** Luca Andreoli, Davide Caputo, Fabio M. Doniselli, Giuliana Messina, Elisa Granocchio, Barbara Castellotti, Elena Freri

**Affiliations:** 1 Department of Pediatric Neuroscience, Fondazione IRCCS Istituto Neurologico Carlo Besta, 20133 Milan, Italy; luca.andreoli@istituto-besta.it (L.A.); elisa.granocchio@istituto-besta.it (E.G.); 2 Department of Humanities and Life Sciences, University School for Advanced Studies, Istituto Universitario di Studi Superiori—IUSS, 27100 Pavia, Italy; 3 Department of Paediatric Neuroscience, European Reference Network EPIcare, Fondazione IRCCS Istituto Neurologico Carlo Besta, 20133 Milan, Italy; elena.freri@istituto-besta.it; 4 Neuroradiology Unit, Fondazione IRCCS Istituto Neurologico Carlo Besta, 20133 Milan, Italy; fabio.doniselli@istituto-besta.it; 5 Unit of Medical Genetics and Neurogenetics, Fondazione IRCCS Istituto Neurologico Carlo Besta, 20133 Milan, Italy; giuliana.messina@istituto-besta.it (G.M.); barbara.castellotti@istituto-besta.it (B.C.)

**Keywords:** *ARFGEF2*, child neurology, epilepsy, periventricular nodular heterotopia

## Abstract

Periventricular nodular heterotopia (PVNH) is a genetically heterogeneous malformation of cortical development with variable neurological outcomes. Among recessive forms, *ARFGEF2*-related disorder is uniquely characterised by the association of diffuse PVNH and progressive microcephaly. We describe a two-year-old boy born to consanguineous parents who presented with severe developmental delay, hypotonia, progressive microcephaly, and infantile-onset epileptic spasms with developmental regression. Brain MRI showed extensive bilateral PVNH associated with callosal hypoplasia and ventriculomegaly. EEG revealed dysmature background activity with multifocal epileptiform discharges and runs of asynchronous fast activity during sleep. Genetic testing identified a novel homozygous nonsense variant in *ARFGEF2*. The clinical course was characterised by drug-resistant epilepsy and multisystemic involvement, including feeding difficulties and recurrent respiratory infections. To contextualise this case, we performed a comprehensive review of previously reported patients, further delineating the clinical, neuroradiological, and electroclinical spectrum of *ARFGEF2*-related disorder. This case highlights progressive microcephaly as a key distinguishing feature of *ARFGEF2*-related PVNH and underscores the importance of early genetic diagnosis to guide targeted surveillance for extra-CNS complications and multidisciplinary care.

## 1. Introduction

Periventricular nodular heterotopia (PVNH) is one of the most common malformations of cortical development, resulting from impaired neuronal migration and occurring either in isolation or in association with other cerebral malformations. It is predominantly associated with genetic aetiologies, with over 20 genes implicated so far [1]. Autosomal recessive periventricular heterotopia with microcephaly [2] was first described in 2003 in two patients and later attributed to biallelic pathogenic variants in *ARFGEF2* [3]. This gene encodes the BIG2 protein, a key regulator of vesicular and membrane trafficking from the trans-Golgi network. These same pathways are disrupted in PVNH associated with other genes, including FLNA and MAP1B [4]. The clinical picture of *ARFGEF2*-related PVNH is characterised by hypotonia, tetraplegia, feeding difficulties, epilepsy, and movement disorders. Extra-CNS manifestations have also been reported, including failure to thrive, recurrent infections, and cardiomyopathy [5]. Although epilepsy is frequent, the electroclinical phenotype has been variably described and requires further characterisation.

We herein describe the clinical, EEG, and MRI phenotype of a patient with PVNH-associated infantile epileptic spasm syndrome (IESS), harbouring a novel homozygous pathogenic variant in *ARFGEF2*. Additionally, we conducted a comprehensive review of previously reported cases to further clarify the phenotypic spectrum associated with this rare condition. The literature review was conducted using the PubMed and Scopus databases with the search term “*ARFGEF2*” for studies published between 1990 and March 2026. The search identified 165 records, of which 92 remained after duplicate removal. Eight articles reporting clinical cases were selected for full-text review; one was excluded because of insufficient clinical information. Two additional relevant articles were identified through forward citation searching using Google Scholar.

## 2. Clinical Case and Literature Review

The proband is a two-year-old male, second child of healthy consanguineous parents (double first cousins) of Pakistani descent. Family history was notable for psychomotor delay in a paternal uncle. The child was born at 41 weeks of gestation via urgent caesarean section due to maternal hypertension, with meconium-stained amniotic fluid but no signs of perinatal distress. Birth parameters were: weight 2800 g (10th percentile), length 50 cm (50th percentile), head circumference 35 cm (25th–50th percentile). Stridor during inspiration was also noted from 3 months, both in wake and sleep.

**Figure 1:**
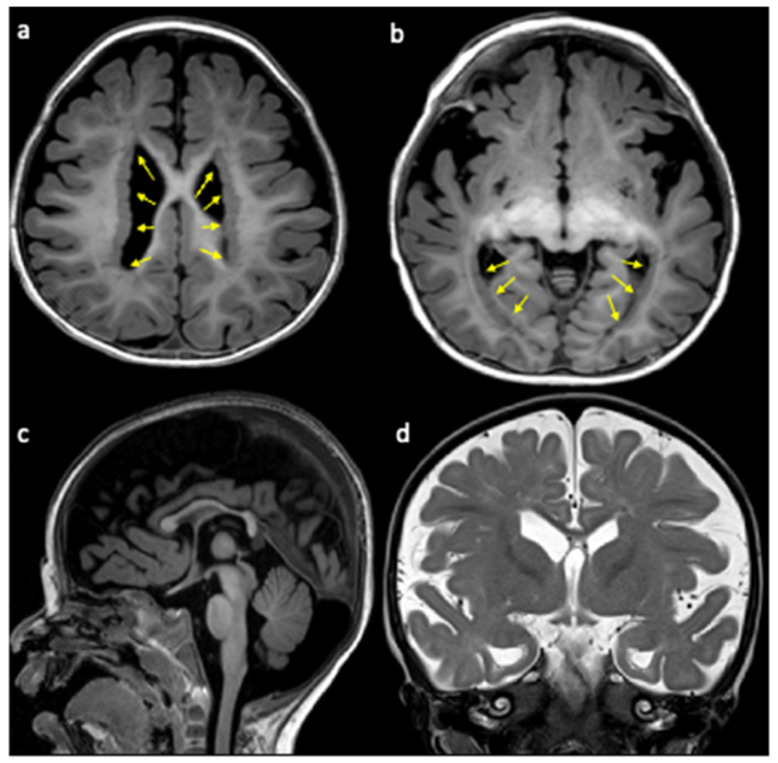
Axial (**a**,**b**) and sagittal (**c**) T1-weighted images and coronal T2-weighted images (**d**), showing reduced brain trophism, diffuse bilateral periventricular nodular heterotopia (yellow arrows), thin corpus callosum (**c**), and dysmorphic and enlarged ventricles.

At first evaluation at 12 months of age, the child exhibited severe psychomotor delay with poor head control and minimal vocalisation. Neurological examination revealed tetraparesis, with hypotonia predominantly affecting the lower limbs. Reduced growth parameters were also observed (weight 7.8 kg, <3rd percentile; height 71 cm, 3rd–10th percentile; head circumference 44 cm, 3rd percentile). Brain MRI revealed reduced brain trophism, extensive bilateral and symmetrical subependymal heterotopic nodules along the walls of the lateral ventricles, hypoplasia of the corpus callosum, dysmorphic and enlarged lateral ventricles, and incomplete eversion of the hippocampi (Figure 1).

**Figure 2:**
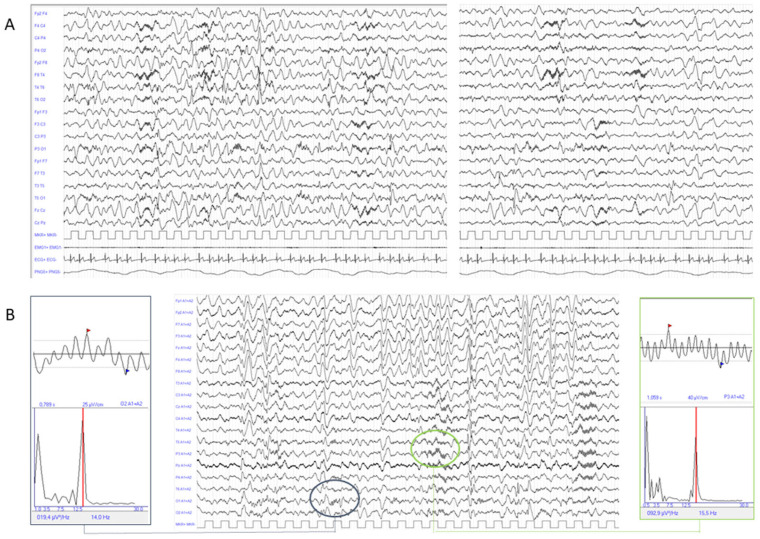
(**A**) The recordings show monomorphic slow wave activity with superimposed sharp waves and sharp and slow wave complexes involving both hemispheres in an asynchronous manner, with a tendency to spread. Fast rhythmic activity at 14–15 Hz is also evident over both centro-temporal and occipital regions. (**B**) EEG displayed in a monopolar montage, with each channel referenced to the left and right mastoid references. Note the characteristic “comb-like” appearance and the frequency overlap with sleep spindles, as indicated by a clear-cut Fourier transform (FFT) peak in the sigma band (**left** and **right** panel). EMG1 = mylohyoid muscle.

At 13 months, the infant developed seizures characterised by transient unresponsiveness, ocular revulsion, and limb extensor spasms, accompanied by crying and moaning. Initially, episodes occurred in clusters during sleep lasting 10–20 min, while later they increased in frequency and also occurred during wakefulness. Concurrently, regression of motor skills was observed, including complete loss of head control. The family sought medical attention at a local centre only when the child was 18 months old, and following an ineffective trial of levetiracetam, the patient was admitted to our institute. EEG recording showed dysmature electrical activity associated with asynchronous epileptic abnormalities over the temporo-parieto-occipital regions bilaterally (see Figure 2A). Moreover, the EEG demonstrated 14–15 Hz rhythmic fast activity over the centro-temporal and occipital regions showing a characteristic “comb-like” appearance and overlapping the frequency range of sleep spindles (see Figure 2B). Visual and auditory evoked potentials were normal. Psychomotor evaluation with Griffiths’ Developmental Scales confirmed profound developmental delay (Developmental Quotient < 20), with scarce response to visual and auditory stimuli, reduced object exploration and manipulation, minimal social interaction, and language limited to rare vocalisations. Progressive microcephaly and moderate dysphagia were also documented. Cardiological evaluation was unremarkable. A diagnosis of IESS was made and vigabatrin was introduced up to a dose of 100 mg/kg, with a slight improvement in seizure frequency and overall condition. The patient’s fragile clinical status due to recurrent respiratory infections prevented the administration of steroid therapy.

A multigene NGS panel containing approximately 500 genes associated with developmental and epileptic encephalopathy (DEE) and cortical malformations was performed. A homozygous nonsense variant in *ARFGEF2* (NM_006420.2): c.2776C > T; p.Arg926Ter was identified. This nucleotide substitution results in the insertion of a premature stop codon in exon 20 of the gene. Based on the current understanding of nonsense-mediated mRNA decay (NMD), transcripts harbouring premature stop codons are typically targeted for degradation. Therefore, this variant is highly likely to result in a loss of function (LoF) through NMD rather than the production of a stable truncated protein. This variant has not been reported in patients or population databases (gnomAD, ExAC, 1000 Genomes). Segregation analysis performed by Sanger sequencing demonstrated that both parents were heterozygous carriers of the variant. Functional studies were not performed but the variant is predicted to result in the production of prematurely truncated protein, and it is classifiable as pathogenic according to the ACMG guidelines (ACMG criteria: PVS1, PM2, PP4, PP1) [6].

A diagnosis of *ARFGEF2*-related IESS was made. An Urdu-speaking mediator was engaged to facilitate communication and ensure the family’s comprehension of the child’s condition, prescribed therapeutic regimen, and feeding recommendations. Genetic counselling was provided to the parents who expressed the desire for future pregnancies. The family was informed about the recurrence risk in future pregnancies and the possibility of prenatal genetic counselling and testing.

From the age of 2 years, the patient experienced global decline with drug-resistant epilepsy despite the addition of clobazam and topiramate, sleep disorder with reduced sleep hours and frequent nighttime awakenings, progressive dysphagia requiring gastrostomy, and recurrent respiratory infections due to food inhalation causing frequent hospitalisation. At last evaluation at 2 years and 10 months, growth parameters were: weight 11 kg (<3rd percentile), height 86 cm (25th–50th percentile), and head circumference 46.5 cm (3rd percentile).

Our comprehensive review of the literature identified 21 individuals (including the present case) with *ARFGEF2*-related PVNH (see Table 1).

## 3. Discussion

We reported a detailed electroclinical and radiological characterisation of a patient with bilateral PVNH and IESS associated with a novel homozygous variant in *ARFGEF2*. Biallelic pathogenic variants in this gene are associated with an autosomal recessive neurodevelopmental disorder characterised by malformations of cortical development and profound psychomotor delay [2,3].

From our literature review no sex predominance was observed, 20/21 patients were born to consanguineous parents, and 17/21 cases occurred in families with more than one affected sibling. All patients carried biallelic variants, with homozygosity documented in 20/21 cases. Reported pathogenic variants include nonsense, frameshift, and splice-site mutations distributed throughout the gene, supporting a LoF mechanism as the primary disease mechanism. No strong genotype–phenotype correlation emerged from the reviewed cases. Most reported cases with truncating variants—whether occurring early or late in the coding sequence—present with broadly similar phenotypes, suggesting that complete loss of function represents the critical pathogenic threshold. Nevertheless, some degree of phenotypic variability has been described, which may reflect modifying factors (such as residual expression, genetic background, or environmental influences) rather than the specific position of the truncation.

While bilateral, diffuse, or contiguous PVNH is a common feature across genetically determined forms, progressive microcephaly represents a key hallmark distinguishing *ARFGEF2*-related conditions, particularly compared with FLNA-associated disease [13]. Functional studies indicate that BIG2 inhibition impairs neural progenitor proliferation and disrupts β-catenin transport, likely contributing to reduced brain growth [4]. Furthermore, a recent genome-wide association study meta-analysis identified *ARFGEF2* as a determinant of early-life head circumference [14]. Additional non-specific malformative features have also been reported, including callosal hypoplasia (14/21), cortico-subcortical atrophy (11/21), white matter and deep grey matter hyperintensities (2/21 and 9/21, respectively), and delayed myelination (3/21).

Common clinical features reported in all cases include severe developmental delay, hypotonia, and tetraparesis. Epilepsy and movement disorders are additional frequent symptoms reported in 13/21 and 10/21 patients, respectively. Extraneurological involvement is also common, including growth impairment (13/21), feeding disorders (10/21) that sometimes require gastrostomy, recurrent respiratory infections, often due to aspiration (8/21), and cardiomyopathy (2/21).

In the literature review, seizures were reported in more than half of patients (13/21, 65%). Although age at onset was not specified, in 7/13 an early onset of seizures seems a common finding. Other than in our case, epilepsy syndrome was reported only in five patients and described as West syndrome evolving to Lennox–Gastaut syndrome in all patients. In the remaining cases only the predominant seizure type was described (three myoclonic seizures and two generalised tonic–clonic seizures). Drug resistance was observed in most cases. Interictal EEG findings were reported in 18/21, including background slowing (7/21), epileptiform discharges (6/21), and hypsarrhythmia (7/21). Despite the limited number of patients described in the literature, our findings could suggest a wide variability in electroclinical presentation in *ARFGEF2*-related epilepsy, similarly to what has been observed in other forms of epilepsy secondary to PVNH [15,16]. The EEG pattern observed in our case is particularly intriguing, as it is characterised by markedly asynchronous bursts of fast “spindle-like” activity over the centro-temporal and occipital regions. This unusual topographic distribution may suggest an immature organisation of fast and slow spindles across the thalamo-cortical network [17]. Alternatively, we hypothesise that it could reflect aberrant network dynamics related to grey matter heterotopia, potentially leading to disrupted synchronisation among cortical neurons. Indeed, heterotopic nodules are known to develop abnormal structural and functional connections with both overlying and distant cortical regions [18].

Furthermore, broader network dysfunction may also be suggested by the presence of movement disorders in half of affected patients, in association with radiological evidence of basal ganglia involvement (atrophy and/or signal abnormalities). This may be explained by the high expression of *ARFGEF2* in the ganglionic eminences, which are critical for basal ganglia development [19]. No basal ganglia alterations were observed in our patient, who did not manifest movement disorders.

Given the rarity of *ARFGEF2*-related disorders and the limited available evidence, the detailed characterisation of our patient, together with a comprehensive review of the literature, adds further insight into the clinical and neuroradiological spectrum of this condition. Moreover, our case contributes a detailed description of the electroclinical phenotype associated with *ARFGEF2*-related epilepsy, an aspect that remains less characterised in previously reported patients.

## 4. Conclusions

*ARFGEF2*-related disorder is a rare genetically determined condition that should be suspected in patients with PVNH and progressive microcephaly. Epilepsy is frequently present, typically showing early onset and heterogeneous electroclinical features, likely reflecting complex network dysfunction related to cortical malformations. Given the high frequency of multisystem complications (cardiac, nutritional, respiratory), early genetic diagnosis is crucial to guide surveillance and ensure coordinated multidisciplinary care. Although therapeutic management of *ARFGEF2*-related epilepsy currently remains phenotype-driven, further understanding of the underlying loss-of-function mechanisms may eventually inform precision medicine approaches and targeted therapies.

## Figures and Tables

**Table 1:** Summary of the literature review of 21 cases of *ARFGEF2*-related periventricular nodular heterotopia (PVNH). BG, basal ganglia; CC, corpus callosum; DD, developmental delay; DEE, developmental and epileptic encephalopathy; GP, globus pallidus; GTCS, generalised tonic-clonic seizures; HC, head circumference; IED, interictal epileptiform discharge; IESS, infantile epileptic spasm syndrome; LGS, Lennox-Gastaut syndrome; LV, left ventricle; MV, mitral valve; NR, not reported; WM, white matter; WS, West syndrome.

	Sheen 2003 [2]		Sheen 2004 [3]		De Wit 2009 [7]	Tanyalcin 2013 [5]			Banne 2013 [8]					Bardon-Cancho 2014 [9]		Yilmaz 2016 [10]	Alojair 2018 [11]		Al Neghery 2018 [12]		Our Case
Patient n°	1	2	3	4	5	6	7	8	9	10	11	12	13	14	15	16	17	18	19	20	21
EPIDEMIOLOGICAL																					
Origin	Turkish	Turkish	Turkish	Turkish	Dutch	Turkish	Turkish	Turkish	Palestinian	Palestinian	Palestinian	Palestinian	Palestinian	NR	NR	Turkish	Saudi	Saudi	Saudi	Saudi	Pakistani
Gender	M	F	F	M	F	M	F	M	M	M	M	F	M	F	M	F	F	F	F	M	M
Consanguinity	+	+	+	+	−	+	+	+	+	+	+	+	+	+	+	+	+	+	+	+	+
Affected sibling	+	+	+	+	−	+	+	+	+	+	+	+	−	+	+	−	+	+	+	+	−
CLINICAL																					
HC at birth	NR	NR	NR	NR	34.2 cm (−1.5 SD)	36 cm	35 cm	36.5 cm	NR	NR	NR	NR	NR	35 cm (75th p.ile)	NR	NR	34 cm	NR	NR (normal)	NR (normal)	35 cm (25–50° p.ile)
HC > 6 m	−2 SD	−2 SD	−2 SD	−2 SD	−2.5 SD	−3.6 SD	−2.4 SD	−3.4 SD	−3/−5 SD	−3/−5 SD	−3/−5 SD	−3/−5 SD	−3/−5 SD	−2 SD	<3° p.ile	<−2SD	−4 SD	−4 SD	NR (micro)	NR (micro)	46.5 cm (3° p.ile)
Reduced growth	+	+	NR	NR	+	+	+	+	NR	NR	NR	NR	NR	+	+	+	+	+	+	NR	+
Age at last examination	2.5 y	16 m	NR	NR	7 m	11 m	9 m	2 y 9 m	NR	NR	NR	NR	NR	3.5 y	15 m	10 y	3 y 5 m	3 y 5 m	3	NR	2 y 10 m
DD	Severe	Severe	Severe	Severe	Severe	Severe	Severe	Severe	Severe	Severe	Severe	Severe	Severe	Severe	Severe	Severe	Severe	Severe	Severe	Severe	Severe
Tetraplegia	+	+	+	+	+	+	+	+	+	+	+	+	+	+	+	+	+	+	+	+	+
Hypotonia	+	+	+	+	+	+	+	+	+	+	+	+	+	+	+	+	+	+	+	+	+
Movement disorder	NR	NR	NR	NR	Dystonia + chorea	Dystonia	−	dystonia	NR	NR	NR	NR	NR	Dystonia	Dystonia+ athetosis	Dystonia+ choreo- athetosis	Dyskinetic	Dyskinetic	Dystonia (upper limbs)	Dystonia (upper limbs)	−
Feeding difficulties	+	+	NR	NR	Gastrostomy/4 y	Gastrostomy/3 y	Gastrostomy/11 m	+	NR	NR	NR	NR	NR	−	−	+	+	+	NR	NR	Gastrostomy/2 y
Vomiting	+	+	NR	NR	+	−	−	+	NR	NR	NR	NR	NR	NR	NR	NR	NR	NR	NR	NR	−
Drooling	NR	NR	NR	NR	+	+	+	+	NR	NR	NR	NR	NR	NR	NR	NR	NR	NR	NR	NR	−
Recurrent infections	+	+	NR	NR	NR	+	+	+	NR	NR	NR	NR	NR	NR	NR	NR	+	+	NR	NR	+
Facial dysmorphisms	NR	NR	NR	NR	−	Low-set ears	−	Low-set ears, low hairline	NR	NR	NR	NR	NR	NR	NR	Bilateral strabismus	NR	NR	−	NR	−
Cardiac involvement	NR	NR	NR	NR	NR	Obstructive cardiomyopathy	NR	NR	−	−	−	−	−	NR	NR	LV non compaction, MV prolapse	NR	NR	NR	NR	−
Age at death	NR	NR	NR	13 y	NR	12 y 9 m	9 y	5 y 11 m	NR	NR	NR	NR	NR	NR	NR	NR	NR	NR	NR	NR	−
EPILEPSY																					
Epilepsy (age at onset)	−	−	+ (Early onset)	+ (Early onset)	−	−	−	+ (9 m)	+ (early onset)	+ (early onset)	+ (early onset)	+ (early onset)	+ (early onset)	+ (18 m)	−	−	+ (6 m)	+ (6 m)	+ (7 m)	+	+ (13 m)
Epilepsy phenotype	/	/	NR	NR	/	/	/	NR	WS → LGS	WS → LGS	WS → LGS	WS → LGS	WS → LGS	Tonic seizures + myoclonic	/	/	GTCS	GTCS	Myoclonic seizures (DEE?)	Myoclonic seizures	IESS
Response to therapy	/	/	Refractory	Refractory	/	/	/	NR	NR	NR	NR	NR	NR	Response to valproate	/	/	NR	NR	NR	NR	Refractory
EEG	Slow background, paroxysmal theta	Dysrhythmic background, occasional SW	Hypsarrhythmia	Hypsarrhythmia	diffuse delta	Diffuse slowing	Slow background	Epileptiform	Hypsarrhythmia	Hypsarrhythmia	Hypsarrhythmia	Hypsarrhythmia	Hypsarrhythmia	Slow BG, diffuse SW	NR	Normal	Slowing + sharp waves in right occipital	Slowing + sharp waves in right occipital	NR	NR	Multifocal IEDs + unusually fast activity
MRI																					
Age at scan	NR	NR	6 m	18 m	4 y 10 m	9 y	9 m	3.5 m	NR	NR	NR	NR	NR	1 y	1 y 3 m	10 y	3 y 5 m	3 y 5 m	3 y	NR	1 y
Cortico-subcortical atrophy	+	+	+	+	+	+	+	+	NR	NR	NR	NR	NR	NR	NR	Fronto-temporal atrophy	WM paucity	WM paucity	NR	NR	−
PVNH	Near-contiguous	Near-contiguous	Contiguous	Contiguous	Near-contiguous	Focal	Near-contiguous	Near-contiguous	Diffuse	Diffuse	Diffuse	Diffuse	Diffuse	Along the ependyma	Diffuse lining temporal horns	Bilateral	Bilateral, cobblestone	Bilateral, cobblestone	+	+	Contiguous
Myelination	NR	Delayed	NR	NR	delayed (13 m)	NR	NR	Delayed	NR	NR	NR	NR	NR	NR	NR	NR	NR	NR	NR	NR	−
WM hyperintensities	NR	NR	T1 hypersignal	NR	−	T2/FLAIR hyperintensity	−	−	NR	NR	NR	NR	NR	NR	NR	NR	NR	NR	−	NR	−
BG trophism/ signal intensity	NR/+	NR	NR	NR	Atrophy/+	Normal/+	Normal/-	Cyst/+	NR	NR	NR	NR	NR	Lenticular atrophy/+ (thalamus)	Lenticular atrophy/+	NR/+ (and caudate)	Putaminal atrophy/+	Putaminal atrophy/+	NR/+ (and GP)	NR	−
Hippocampus	NR	NR	NR	NR	Mild atrophy	Prominent atrophy	Prominent atrophy	Atrophy	NR	NR	NR	NR	NR	NR	NR	NR	NR	NR	NR	NR	Incomplete eversion
CC	NR	Thin	Small (post>ant)	NR	NR	Small (CC 4 cm)	Small (CC 2 cm)	Thin and short (3.5 cm)	Thin	Thin	Thin	Thin	Thin	Thin	Thin (post > ant)	NR	Thin (body and splenium)	Thin (body and splenium)	Normal	NR	Hypoplasia
Brainstem/cerebellum	NR/ normal	NR/abnormal posterior fossa	Normal	NR	NR	Normal	Normal	Normal	NR	NR	NR	NR	NR	NR	NR	NR	NR	NR	NR	NR	−
GENETICS																					
*ARFGEF2* variant	c.625G > A p.Glu209Lys	c.625G > A p.Glu209Lys	c.242_249 delins7	c.242_249 delins7	c.2031_2038dup c.3798_3802del	c.242_249 delins7	c.242_249 delins7	c.242_249 delins7	c.1958 (+1) G > A	c.1958 (+1) G > A	c.1958 (+1) G > A	c.1958 (+1) G > A	c.1958 (+1) G > A	c.388 C > T	c.388 C > T	c.5126 G > A	c.1958 (+ 1) G > A	c.1958 (+ 1) G > A	c.3974 G > A p.Trp1325Ter	c.3974 G > A p.Trp1325Ter	c.2776 C > T p.Arg926Ter
Homozygosity	+	+	+	+	−	+	+	+	+	+	+	+	+	+	+	+	+	+	+	+	+
Mutation type	Missense	Missense	Frameshift	Frameshift	Frameshift	Frameshift	Frameshift	Frameshift	Splicing	Splicing	Splicing	Splicing	Splicing	Nonsense	Nonsense	Nonsense	Splicing	Splicing	Nonsense	Nonsense	Nonsense

## Data Availability

The original contributions presented in this study are included in the article. Further inquiries can be directed to the corresponding author.

## Abbreviations

The following abbreviations are used in this manuscript:

EEG	Electroencephalography
IESS	infantile epileptic spasm syndrome
MRI	Magnetic Resonance Imaging
NMD	Nonsense-Mediated Decay
PVNH	periventricular nodular heterotopia
